# Construction and verification of the targeted uPA-shRNA lentiviral vector and evaluation of the transfection and silencing rate

**DOI:** 10.3892/etm.2014.1741

**Published:** 2014-05-28

**Authors:** WEI-SHAN WANG, FENG-JING GUO, CHANG-JUN LI, ZHEN-DONG ZHANG, CHEN-HUI SHI

**Affiliations:** 1 Department of Orthopedics, Medical College of Shihezi University, Shihezi, Xinjiang 832008, P.R. China; 2 Department of Orthopedics, Tongji Hospital, Tongji Medical College, Huazhong University of Science and Technology, Wuhan, Hubei 430030, P.R. China

**Keywords:** urokinase-type plasminogen activator, RNA interference, lentiviral vector, osteoarthritis, chondrocytes

## Abstract

Urokinase-type plasminogen activator (uPA) receptors, which are released by the synovial tissue, are responsible for the activation of cartilage-breakdown proteases and play critical roles in cartilage degradation during the progression of osteoarthritis (OA). RNA interference (RNAi) technology has emerged as a potent tool to generate cellular knockdown phenotypes of a desired gene. The aims of the present study were to investigate the effect of siRNA specific to the uPA gene on chondrocytes and to investigate the possible mechanisms of OA. Firstly, four types of small hairpin RNA (shRNA) sequence (P1, P2, P3 and P4) were obtained from the targeted uPA gene of the New Zealand rabbit, based on siRNA theory. The sequences were designed, constructed and subjected to restriction enzyme digestion, transformation, polymerase chain reaction (PCR) identification, positive clone sequencing and lentivirus packaging. Secondly, primary culturing cartilage cells from the New Zealand rabbit were transfected with P1, P2, P3 or P4 to observe the transfection rate under a fluorescence microscope. The mRNA expression levels of uPA were analyzed in cartilage cells using quantitative PCR, while protein expression levels were analyzed in the cartilage cells using western blot technology. Four types of uPA-shRNA lentiviral vectors were constructed successfully, which were all able to be transfected into the primary culturing cartilage cells. The transfection rate was as high as 85% when the multiplicity of infection was 100, which demonstrated that P1, P2, P3 and P4 were all capable of inhibiting the mRNA and protein expression of uPA in cartilage cells. In addition, among the four sequences, the P2 sequence exhibited the highest silencing rate of 70%. Statistical significance (P<0.05) was observed when analyzing the silencing rate of P2 compared to the other three groups. The most efficient targeted uPA-shRNA sequence was identified following screening. The results strongly verified that siRNA lentiviral vectors can be transfected into cartilage cells to further inhibit the expression of the uPA gene efficiently and steadily. Thus, the results provide the foundation for further research on the role of uPA in the pathogenesis of OA.

## Introduction

Urokinase-type plasminogen activator (uPA) is one of the most important family members of the serine proteolytic enzymes (1). In the human body, uPA combines with the uPA receptor under physical and pathological conditions, affecting cell migration, tissue reparation and mediating the hydrolysis of extracellular matrix proteins, the degradation of collagenase and the activation of other proteolytic enzymes, while also directly degrading the extracellular matrix and basilar membrane (2). uPA has also been shown to be highly associated with the invasion and metastasis of tumors and the inflammatory reaction of the synovium. A recent study demonstrated that the serine protease plays an important role in matrix metalloproteinase (MMPs)-mediated degradation of cartilage tissue in arthritis (3). Although uPA is an important member of the serine protease family, the mechanisms underlying uPA function in the MMP signaling transduction pathway remain unknown. Post-transcriptional gene silencing is known as RNA interference technology (RNAi) and can be used to specifically inhibit the regulation of gene expression following transcription. This new method is used for quick silencing of one specific gene (4). To date, the technique has been widely applied in functional genomes and gene therapy. In the present study, a lentiviral vector was constructed to specifically target the uPA gene in the New Zealand rabbit. The aim of the study was to establish the foundation for further research on the role of uPA in the pathogenesis of osteoarthritis (OA).

## Materials and methods

### Materials and reagents

A total of 3 New Zealand rabbits (age, 2 years) were purchased from the Experimental Animal Center of Xinjiang Medical University (Ürümqi, China). *Escherichia coli (E. coli)* DH5α and 293T cells had been preserved in the research center. The lentivirus-negative control (NC) and pGLV-H1-green fluorescent protein (GFP)+Puro vector were purchased from Zimmer Medical International Trading Co., Ltd. (Shanghai, China). The restriction enzymes, *Bam*HI and *Eco*RI, were obtained from MBI Fermentas (Amherst, NY, USA). The transfection reagent, Lipofectamine 2000, as well as TRIzol reagent and Dulbecco’s modified Eagle’s medium/F12 culture medium were purchased from Invitrogen Life Technologies (Carlsbad, CA, USA). The *Taq* enzyme and plasmid extraction kit were obtained from Takara Bio, Inc. (Shiga, Japan), while the uPA antibody was purchased from Abcam (Cambridge, MA, USA). All animals were maintained in the Animal Facility of the Shihezi University School of Medicine. The experimental protocol was reviewed and approved by the Institutional Animal Care and Use Committee of the Shihezi University, Shihezi, China.

### Primer synthesis

Primers for uPA and the β-actin reference were designed using Oligo 6.0 software (Molecular Biology Insights, Inc. Cascade, CO, USA), which was conducted by Shanghai Sangon Biological Engineering Technology & Services Co., Ltd. (Shanghai, China). The sequences of the primers were as follows: uPA upstream, 5′-ACTACATTG TCTACCTGGGTCGGTC-3′ and downstream, 5′-ATGCAA GATGAGTTGCTCCACTTC-3′ (amplification length, 86 bp); β-actin upstream, 5′-CAGGTCATCACCATCGGCAAC-3′ and downstream, 5′-GGATGTCCACGTCGCACTTCA-3′ (amplification length, 133 bp).

### Cell culture

Cartilage tissue was obtained from the knee-joint of a two-year-old New Zealand rabbit. The cartilage cells were collected from cartilage tissue by repeated digestion for four times, then cultured at flask with culture medium at 37°C in a 5% CO_2_ humidified incubator. The medium was changed every two days. Cells were observed under an inverted microscope and images and observations were recorded with regard to the cellular morphology and adherence conditions. Further experiments were conducted when the cells are 85–90% in a confluent state for later use.

### Designing and synthesizing uPA-siRNA

Using GenBank, the uPA gene sequence in rabbits was identified (NM-001082011). Referring to the design principles of the targeted point of siRNA, four targeted points were selected and designed using GenePharma siRNA designer v 3.0 software (Shanghai GenePharma, Shanghai, China), which were P1, P2, P3 and P4 (Table I). A pervasive disturbing sequence was also designed as the NC. The structure of the small hairpin RNA (shRNA) chain was positive-sense strand-loop-antisense strand. Each group of sequences were designed and synthesized according to the hairpin structural model. Restriction enzyme digestion was conducted at the two ends of the molecules, thus, the specific gene sequence was inserted into the vector directly following digestion (Table II). The sequencing fragments were synthesized by Zimmer Medical International Trading Co., Ltd (Shanghai, China).

### Interference lentiviral vector construction

**Figure 1:**
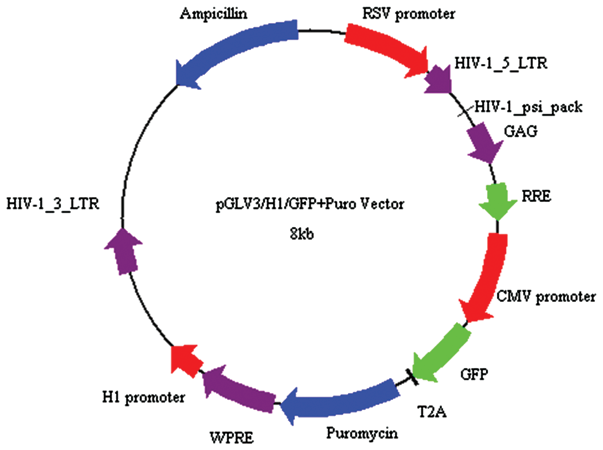
pGLV-Hl-GFP+Puro plasmid map. GFP, green fluorescent protein.

As shown in Table II, following the dilution of the oligonucleotide fragments, double stranded DNA fragments were formed in the annealing reaction system. The pGLV-HI-GFP+Puro vector was linearized by *Bam*HI and *Eco*RI restriction enzyme digestion (Fig. 1). Pure linearized vector fragments, double stranded DNA fragments and vector fragments were collected and combined together during a 12-h reaction. The recombinant vector loop was then transformed into the freshly prepared *E. coli* competent cells. Following *E. coli* cell culture for 16 h at 37°C, bacterial colonies were selected randomly as polymerase chain reaction (PCR) templates. Verification of the positive clones was conducted using PCR technology. The sequencing fragments were synthesized by Shanghai Sangon Biological Engineering Technology & Services Co., Ltd.

### Recombinant lentiviron packaging and titering

Using uPA genes, RNAi lentiviral vectors and pMD2.G plasmids were tranfected together in 293T cells. After 8 h, the culture medium was changed to complete medium. The supernatant was concentrated and collected after culturing for 48 h. The virus titer of the 293T cells was determined using the dilution gradient method and calculated as follows: Virus titer (TU/ml) = (counted fluorescent cells/corresponding dilution times)/0.01. The transfected cells were stored in a −80°C refrigerator for later use.

### Examination of cell transfection and the transfection rate

Packaged recombinant lentivirons were classified into three groups based on multiplicity of infection (MOI) values (1, 10 or 100), and were transfected into the cartilage cells separately. The culture medium was changed to complete medium at 6 h following transfection. Cells were cultured for 3 days, following which the expression level of GFP in the cells was observed using an inverted fluorescence microscope. The transfection rate was also analyzed using Image-Pro Plus 6.0 software (Media Cybernetics, Inc., Rockville, MD, USA).

### Quantitative PCR (qPCR)

Total RNA was extracted at day 4 following transfection using TRIzol reagent. Reverse transcription of the RNA to cDNA was conducted using the RNA as a template, and then PCR-amplification of the uPA gene was performed using the cDNA as a template. The primers were designed by Shanghai Sangon Biological Engineering Technology & Services Co., Ltd. The qPCR conditions were as follows: Primary degeneration for 15 sec at 95°C, degeneration for 5 sec at 95°C and annealing for 30 sec at 60°C, which was repeated 40 times. Light absorption values were assayed at each extension stage. Following PCR, degeneration of the PCR product was conducted for 1 min at 95°C, which was then cooled-down to 55°C in order for the DNA double strands to combine together fully. A melting curve was constructed using the light absorption values.

### Western blot analysis

Total protein was extracted at day 4 following transfection and gel-electrophoresis was conducted using 10% SDS-PAGE following protein quantification. Next, the proteins were transferred to a nitrocellulose membrane and then incubated with primary antibodies including PLAU (1:5,000 dilute, GeneTex Inc., Irvine, CA, USA) and β-actin (1:10,000 dilute. Abcam Inc., Shanghai, China) overnight at 4°C followed with anti-rabbit second antibodies (1:10,000 dilute, GeneTex Inc.) at room-temperature for one hour. Enhanced chemiluminescence was used for detection. β-actin was used as the internal reference.

### Statistical analysis

All the results are expressed as the mean ± standard deviation. Data were analyzed by single factor analysis of variance and the t-test using SPSS 13.0 software (SPSS, Inc., Chicago, IL, USA). P<0.05 was considered to indicate a statistically significant difference.

## Results

### Vector construction and verification of the positive clone using PCR

**Figure 2:**
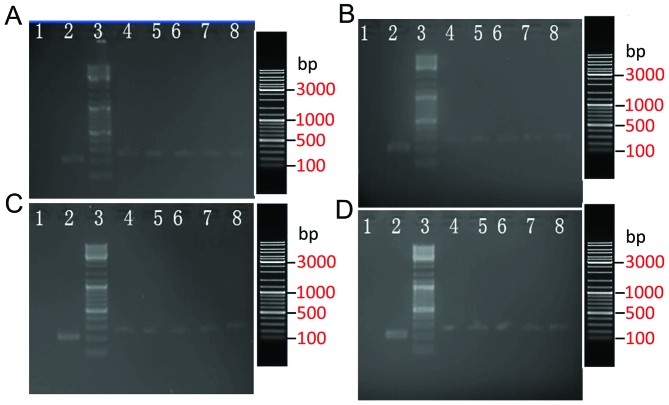
Electrophores of (A) P1, (B) P2, (C) P3 and (D) P4 sequence vectors and PCR products. Lanes: 1, ddH_2_O NC; 2, control; 3, markers (19.3, 7.7, 6.2, 4.2, 3.4, 2.6, 1.8, 1.4 and 0.9 Kb); 4–8, positive colony group. PCR, polymerase chain reaction; NC, negative control.

The size of the PCR amplification product, which was from the positive clone of the inserted shRNA sequence, was 384 bp according to the primer size. By contrast, the size of the PCR amplification product for the empty vector clone was 322 bp (Fig. 2). These results demonstrated that all five random clones were positive clones. The sequencing results of the shRNS carrier vector revealed the expected sequence, indicating that the shRNA vector was constructed successfully. DNA sequencing further demonstrated that the sequence was correct and showed no mutations.

### Lentivirus packaging and viral titer

**Figure 3:**
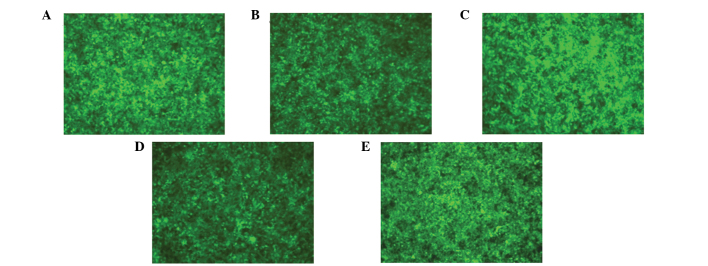
Expression of GFP in 293T cells transfected with the (A) P1, (B) P2, (C) P3, (D) P4 and (E) NC sequence vectors (immunofluorescence; magnification, ×200). GFP, green fluorescent protein; NC, negative control.

293T cells were transfected using the successfully constructed uPA RNAi lentiviral vectors and pMD2.G coated plasmids. Significant expression of GFP was observed under the fluorescence microscope after 40 h (Fig. 3), and the biological titer for the collected and concentrated virus was 1×10^8^ TU/ml, as determined by the hole dilution method.

### Examination of the lentivirus transfection rate

**Figure 4:**
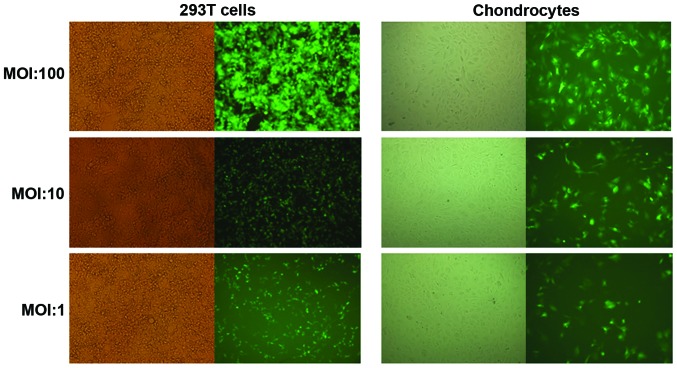
Transfection efficiency of the P3 lentivirus vector with different MOI values in 293T and chondrocyte cells after 72 h (immunofluorescence; magnification, ×200). MOI, multiplicity of infection.

Transfection rates of the four lentiviral vectors in 293T cells and cartilage cells were observed under the various MOI values. The results demonstrated that with increasing MOI, the lentivirus transfection rate also improved. When the MOI was 100, the transfection rate was >85% (Fig. 4), which was which was used for the following experiments.

### Examination of the interference rate of uPA-siRNA

**Figure 5:**
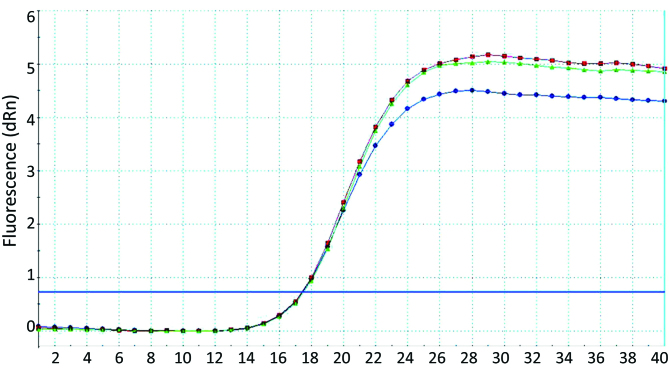
qPCR amplification curve. qPCR, quantitative polymerase chain reaction.

**Figure 6:**
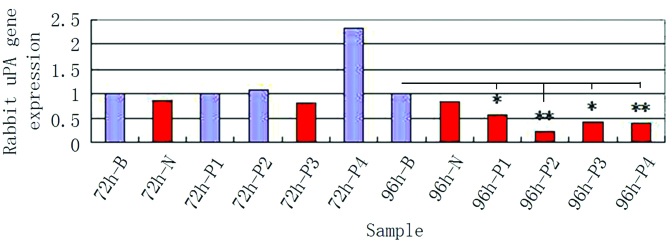
mRNA expression levels of uPA following transfection. ^*^P<0.05 and ^**^P<0.01, P1, P2, P3, P4 vs. 96-h B.

**Figure 7:**
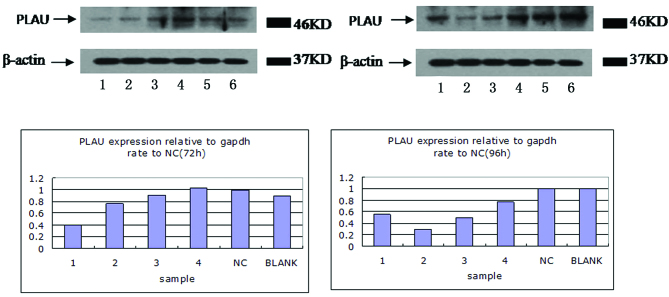
Protein expression levels of uPA following transfection for 72 and 96 h. 1, P1; 2, P2; 3, P3; 4, P4; 5, NC; and 6, blank. NC, negative control; uPA, urokinase-type plasminogen activator.

Relative qPCR was performed with β-actin as the internal reference and the results were analyzed using the standard curve method. The results indicated that the mRNA expression level of uPA decreased significantly following transfection of P1, P2, P3 or P4 plasmids into the cartilage cells (Fig. 5). In addition, the corresponding rates for the empty plasmid and NC increased, further demonstrating that these four plasmids exhibited a significant inhibiting influence on uPA (Fig. 6). Furthermore, the knockout effectiveness for the P2 RNAi plasmid was better compared with the other three plasmids. This observation was confirmed by western blot analysis. The four plasmids all inhibited the protein expression of uPA, however, the knockout effectiveness for P2 and P3 RNAi plasmids was better compared with the P1 and P4 RNAi plasmids (Fig. 7; P<0.05). Therefore, the P2 plasmid may have the most efficient inhibiting effect on uPA.

## Discussion

OA is a progressive degenerative disease with the main pathological feature of mechanical abnormalities in the cartilage tissue (5). Although there have been a number of comprehensive clinical and fundamental studies, the definite pathomechanism underlying the pathological changes remains unclear, thus, clinical treatment is becoming more difficult (6–8). In general, OA is considered to be a chronic degenerative disorder, involving all areas of the joint, including cartilage tissue, subchondral bone and the synovium (9). OA may be caused by a combination of multiple factors, including tissue damage, supersession, metabolization, genetics, heredity and immunization (10,11). Based on a number of in-depth studies that used molecular biological technology, it has been identified that MMPs, uPA, A disintegrin and metalloproteinase with thrombospondin motifs, among other proteolytic enzymes, are the most fundamental factors contributing to the degradation process of cartilage tissue (12–16). In addition, the uPA that is secreted by macrophages in the synovium has been shown to play an important role in the primary stages of OA, by causing the synovial tissue to secrete large amounts of interleukin-1β, which in turn is involved in increasing the synovial inflammatory reaction and the degradation process. Furthermore, uPA can directly affect the degradation of the extracellular matrix in cartilage cells and activate zymogens of MMPs, transforming them into active protein degradation enzymes that upgrade the degradation of the extracellular matrix in cartilage cells. In addition, uPA is involved in the regulation of mitogen-activated protein kinase and other signaling pathways, thus, can influence the metabolic process of cartilage cells (3,17,18). Therefore, reducing the early degenerative metabolic process of cartilage tissue can be achieved by inhibiting the expression of uPA in the synovial and cartilage tissues.

RNAi is a biological process that causes specific homologous mRNA degradation following internal or external double stranded RNA entering the cells. The technique inhibits the expression of the corresponding gene, which is followed by the phenomenon of specific gene deletion. RNAi technology was developed based on this model and has become a newly developed biotechnology in recent years. The technique is a much simpler, more efficient and powerful tool compared with gene knockout, and plays an important role in gene functioning and gene treatment. Numerous studies have shown that RNAi inhibits gene expression definitely, and even applying traces of siRNA can decrease the coded pathogenic gene production by 90% (19–21). Furthermore, RNAi can knockout all the specific genes during an experiment (22,23). Due to the cascading amplification effect and the highly penetrative characteristic of RNAi, its usage has a significant prospect in gene functional research and gene treatment. However, previous biochemistry and adenoviral vector methods resulted in inefficient and unstable transfection, thus, successful transfection of the RNAi sequence into the original targeted cells has become a barrier for the application of this procedure. However, as a result of the recent identification of the lentiviral vector, the transfection rate can reach 70% and gene expression is stable for a long time in cells during cell tranfection. In order to assess the effect of uPA in gene treatment for the early pathological changes of OA, in the present study, a specific targeted lentiviral vector was successfully constructed against the uPA gene in a New Zealand rabbit. The targeted sequence, which can highly silence the uPA gene, was screened out, thus, this study established the foundation for further research on the role of uPA in the pathogenesis of OA.

In conclusion, based on the uPA gene, the four designed shRNA recombinant lentiviral vectors were all shown to accomplish gene interference in the original cartilage cells of the New Zealand rabbit. Expression levels of the targeted uPA gene prior to and following silencing were compared using qPCR, and differences in the silencing effectiveness were identified among the four types of shRNA recombinant lentiviral vectors. Silencing effectiveness was closely associated with the targeted sequence and virus titer. The more concentrated the vectors during transfection, the better the silencing effect. The combined results from qPCR and western blot analysis revealed that P2 specific targeted-shRNA sequence exhibited significant silencing efficacy. The results of the present study have established the foundation for using this targeted point to improve the research on the application of uPA in OA development and gene treatment.

## Figures and Tables

**Table I:** Target sequences for the uPA gene.

Vector	Gene locus	Effect	Sub-sequence
P1	PLAU-Oc-795	Interference target gene	GCGCCACACATTGCTTCATTA
P2	PLAU-Oc-855	Interference target gene	GGTCAAGGCTTAACTCCATGA
P3	PLAU-Oc-901	Interference target gene	GGAGCAACTCATCTTGCATGA
P4	PLAU-Oc-676	Interference target gene	GGGAGAATTCACCATCATTGA
NC	/	Negative control	UUCUCCGAACGUGUCACGUTT

NC, negative control; uPA, urokinase-type plasminogen activator.

**Table II:** Design and synthesis of uPA shRNA targeted sequences.

Vector	Sense	5′	STEMP	LOOP	STEMP	3′
P1	S	GATCC	GCGCCACACATTGCTTCATTA	TTCAAGAGA	TAATGAAGCAATGTGTGGCGC	TTTTTTG
	AS	AATTCAAAAAA	GCGCCACACATTGCTTCATTA	TCTCTTGAA	TAATGAAGCAATGTGTGGCGC	G
P2	S	GATCC	GGTCAAGGCTTAACTCCATGA	TTCAAGAGA	TCATGGAGTTAAGCCTTGACC	TTTTTTG
	AS	AATTCAAAAAA	GGTCAAGGCTTAACTCCATGA	TCTCTTGAA	TCATGGAGTTAAGCCTTGACC	G
P3	S	GATCC	GGAGCAACTCATCTTGCATGA	TTCAAGAGA	TCATGCAAGATGAGTTGCTCC	TTTTTTG
	AS	AATTCAAAAAA	GGAGCAACTCATCTTGCATGA	TCTCTTGAA	TCATGCAAGATGAGTTGCTCC	G
P4	S	GATCC	GGGAGAATTCACCATCATTGA	TTCAAGAGA	TCAATGATGGTGAATTCTCCC	TTTTTTG
	AS	AATTCAAAAAA	GGGAGAATTCACCATCATTGA	TCTCTTGAA	TCAATGATGGTGAATTCTCCC	G
NC	S	GATCC	UUCUCCGAACGUGUCACGUTT	TTCAAGAGA	AAACGTGACACGTTAGGAGAA	TTTTTTG
	AS	AATTCAAAAAA	UUCUCCGAACGUGUCACGUTT	TCTCTTGAA	AAACGTGACACGTTAGGAGAA	G

NC, negative control; uPA, urokinase-type plasminogen activator; S, sense; AS, antisense.
